# The Internet of Things for the Intelligent Management of the Heating of a Swimming Pool by Means of Smart Sensors

**DOI:** 10.3390/s23052533

**Published:** 2023-02-24

**Authors:** Álvaro de la Puente-Gil, Miguel de Simón-Martín, Alberto González-Martínez, Ana-María Diez-Suárez, Jorge-Juan Blanes-Peiró

**Affiliations:** Energy Resources’ Smart Management (ERESMA) Research Group, Escuela Superior y Técnica de Ingenieros de Minas, Universidad de León, 24071 León, Spain

**Keywords:** smart sensors, internet of things, intelligent management, power consumption

## Abstract

By using various smart sensors integrated in a global domotic system, a proper solar thermal management is executed. The goal is to properly manage solar energy for heating swimming pool using various devices installed at home. Swimming pools are a necessity in many communities. In summer, they are a source of refreshment. However, maintaining a swimming pool at an optimal temperature can be a challenge even in the summer months. The use of the Internet of Things in homes has enabled proper management of solar thermal energy, thus significantly improving the quality of life by making homes more comfortable and safer without using additional resources. The houses built today have several smart devices that manage to optimize the energy consumption of the house. The solutions proposed in this study to improve energy efficiency in swimming pool facilities include the installation of solar collectors to heat swimming pool water more efficiently. The installation of smart actuation devices (to efficiently control energy consumption of a pool facility via different processes) together with sensors that provide valuable information on energy consumption in the different processes of a pool facility, can optimize energy consumption thus reducing overall consumption (by 90%) and economic cost (by more than 40%). Together, these solutions can help to significantly reduce energy consumption and economic costs and extrapolate it to different processes of similar characteristics in the rest of the society.

## 1. Introduction

Energy is a vital source of energy used in a variety of productive and service sectors [1] and also in the domestic sector [2]. Countries depend on energy in their economic growth process; without it, their economies would slow down or even stop altogether. Without energy, businesses cannot produce goods and governments cannot maintain social programs. Energy is even more important for developing countries.

On the other hand, climate change is one of the most serious problems facing humanity today. According to the U.S. Oceanic and Atmospheric Administration (NOAA), during 2019, the planet reached the highest average temperature value since data has been available.

In the field of energy efficiency, it is important to highlight the commitment made in 1990 by the Member States of the European Union (EU) to reduce energy consumption and CO_2_ levels 20% of the values of the year 2020, a target that is far from being achieved. In this context, growth in global energy consumption slowed in 2019 (+0,6%), down from its previous trend of 2% [3].

How countries consume and produce energy has become an important issue in world politics. Some nations rely on exporting goods and services to generate income, while others seek to import energy from external sources. Exporting countries require the cooperation of other countries to ensure that they do not run out of energy resources.

Focusing on Spain, energy consumption, as in the rest of the world, is based mainly on fossil energy sources, oil, and natural gas. It is worth noting the high dependence on oil, of which we import more than 99% [4]. In Spain, the Climate Change and Energy Transition Law and its corresponding corrections have already been published [5,6].

According to the International Energy Agency [7], an average household in Spain consumes around 3000 kilowatt-hours of electricity per year, which is lower than the average in most other European countries. Since 2000, Spain has reduced its average annual electricity consumption by 3% [8].

Despite this, it is important to keep in mind that energy storage currently has its limits. The country with the highest energy consumption per capita is Germany, followed by Russia and Japan [9]. In these countries, people have high expectations for their standard of living. Fortunately, there are ways to reduce the amount of energy we consume, but we must start implementing those changes now to avoid serious consequences in the future. Spain has done a good job reducing its energy consumption over time. Between 1980 and 2013, its average annual electricity consumption decreased by 21%. In addition, its gas consumption decreased by 31% during this time period [9]. This reduction demonstrates that it is possible to reduce resource consumption without suffering major setbacks in quality of life. However, we must continue to work to reduce our consumption to avoid significant ecological damage. There are many ways in which excessive energy consumption damages the environment. For example, burning fuel releases carbon dioxide into the atmosphere, which is harmful to the environment. Fortunately, there are alternatives to reduce these problems; however, there are still many sources of pollution in our environment. In conclusion, Spain has shown that it is possible to reduce its country’s energy consumption without sacrificing its quality of life.

**Figure 1:**
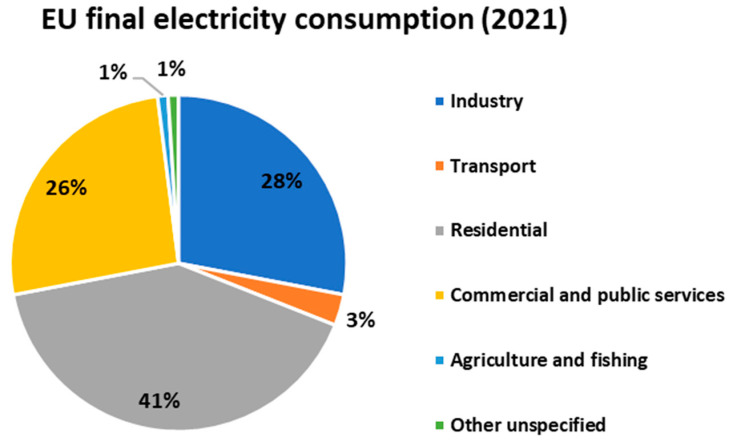
Final energy consumption European Union (EU) in 2021 [7].

Figure 1 shows the distribution of electricity consumption in Europe in the different sectors [7].

### 1.1. State-of-the-Art

The continuous increase in the demand for electricity is causing problems in the power grid and has prompted the development of alternative energy sources. As a result, many countries are converting their existing infrastructure to accommodate these new energy sources.

Household electricity consumption includes uses as diverse as electronics, lighting, cooling and heating systems, and household appliances [10]. Although household consumption is increasing, there are several ways to control it. It can be reduced, for example, by limiting the number of lights and replacing them with energy-saving ones. It is important to keep in mind that limiting these uses of electricity may affect comfort levels [11]; however, it can ensure that significant savings are realized without compromise.

Technology has changed our lives in countless ways, but it also brings challenges. Modern technology includes mobile devices and the Internet. Many devices today come with online connectivity capabilities [12]. Any connected device can now access the web for configuration and programming [13].

Home automation is the concept of combining electronic devices with everyday tasks to create a more convenient home environment. It helps to ease the burden of housework, and makes life more comfortable. A home automation system provides control over the home without the need to physically be there. This technology can be used to solve common home management problems although it may have security vulnerabilities [14].

Home automation has many advantages over manual tasks. Each system can be configured to suit the user’s needs and preferences. Actually, the temperature, light levels, lighting levels [15] and music selections in different parts of the home can be controlled. It also allows to set up routines for daily tasks such as feeding the pets or making coffee [16].

There are several types of home automation systems [17]. The most common consists of a computer network that controls various appliances and electrical systems within the home. These systems include security devices [18], lights, temperature, appliances, entertainment, and more. Another type of system connects items inside the home to the Internet so they can be easily accessed and controlled from anywhere using a cell phone or tablet. There are also systems that automatically send notifications when a device is damaged within the home. This allows to address problems quickly without tedious searches for specific items [19].

Home automation is easy to customize according to the user’s needs and preferences. Functionality can be added by incorporating additional devices or by generating software implemented on a computer, tablet or phone [20]. There are also many different projects to build circuit boards, software, sensors, switches, controllers, and more using different materials such as Arduino, Raspberry Pi and Beagle Bone Black kits [21,22]. After building the system, it is possible to control all aspects of an automated home from anywhere in the world.

There are many benefits to adding home automation devices and accessories [23] but it is convenient not to necessarily have to purchase new hardware when adding new devices to the system. It is possible to configure routines for daily tasks without the need for any additional equipment [24].

Several works have been developed to search for intelligent energy management applications in domestic and industrial environments [25]. In addition, there are systems that allow the integration of vehicle recharging in the domestic sector by performing a management of the recharging process [26]. These developments cover a wide range of applications, for example, from the use of techniques to estimate the energy consumption of the house according to different characteristics (seeking to optimize the energy demand in a home [27,28]) up to management of irrigation water consumption with smart systems [29].

### 1.2. Contribution and Structure of the Article

Pool water heating is an important task for any pool owner located in moderately cold geographical areas. This can be a highly energy consuming task. The heating system and the cost associated with it is one of the primary issues that must be addressed in the design phase of the swimming pool.

To get a rough idea of the figures, according to a report prepared by the Spanish Association of Swimming Pool Professionals (ASOFAP). Spain is the fourth country in the world and the second in Europe in number of swimming pools for residential use, with an estimated 1,018,000 units in 2018. Adding the pools for residential, public, and collective use, the number of pools in Spain amounts to almost 1,2 million units, which corresponds to one pool for every 39 citizens.

For its part, the Spanish Association of Swimming Pool Professionals (ASEPPI) estimates that privately owned swimming pools in Spain have an average capacity of 40 cubic meters, while those for public use have an average of 200 cubic meters. With these data, it is calculated that the aquatic facilities of the country have a total capacity of 62,400,000 cubic meters. For that reason, to have a sustainable swimming pool, so much economic, as ecologically it represents a task of obligatory fulfillment.

Regarding the technology used for heating, some owners chose the electricity. However, this is inefficient and costly. Other times, solar panels are used, but this solution is not always possible and involves proper energy management.

This paper compares different scenarios in which the heating of a private swimming pool for domestic use is managed with different alternatives, combined with the pool purification time. The objective is to implement optimization algorithms to perform the processes of heating the water and purifying the pool on a daily basis according to the information received from smart sensors.

Section 2 explains the different equipment used and the scenarios considered. Section 3 shows the results obtained and finally Section 4 develops the conclusions.

## 2. Materials and Methods

This section details the devices and the different scenarios with the diverse heating options for a particular domestic swimming pool. The pool in question has a volumetric capacity of 47 m^3^. The heating process has to raise the temperature of the pool water from 25 °C, which is reached during the night hours, to a comfortable 27 °C for the swimming daylight hours.

**Figure 2:**
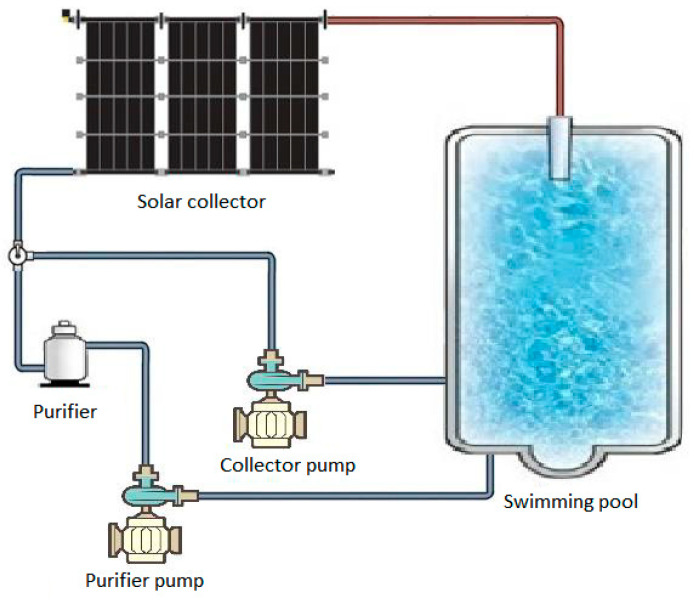
Schematic diagram of the existing components of the installation.

**Figure 3:**
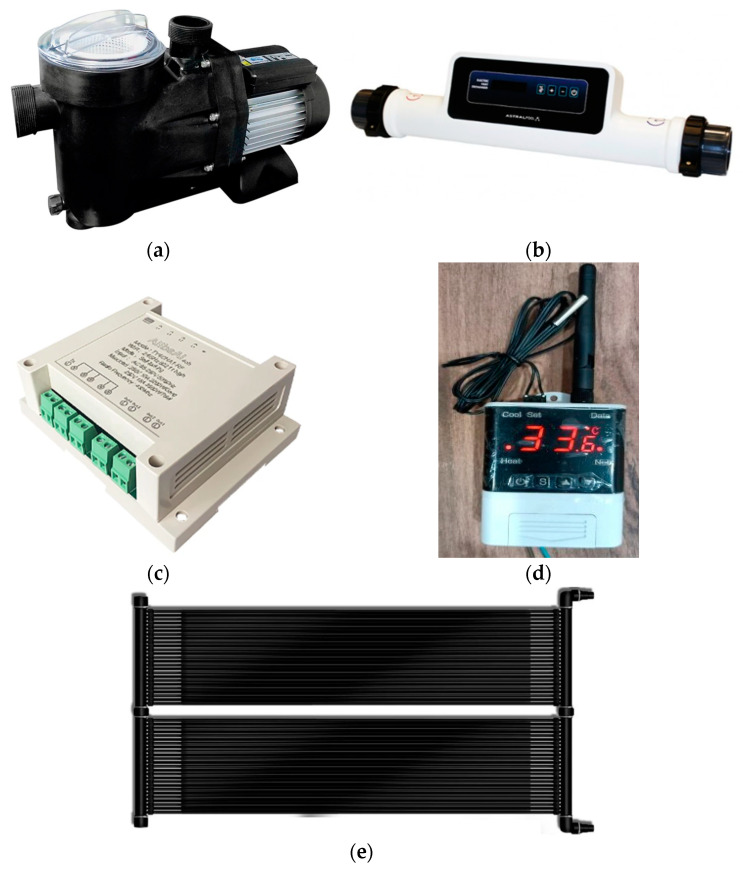
Devices used (**a**) Hydraulic pumps. (**b**) Electric heater. (**c**) 4-channel relay. (**d**) Smart temperature sensor. (**e**) Solar collector.

Figure 2 shows the connection scheme of the different components of the installation that is shown in Figure 3.

Algorithms are developed based on the temperatures provided by smart sensors installed in the solar collectors and in the swimming pool itself.

### 2.1. Description of the IoT Management System Used

The home automation manager selected for the operation of the various smart devices in the system is the Home Assistant. This is an open source home automation system that can be used to control a wide variety of devices in the home. It allows users to control and automate their lighting, heating, security and more from one easy-to-use interface. In addition, Home Assistant is compatible with a wide variety of home automation devices and systems, allowing users to create a customized automation system for their specific needs. The users can control their home’s devices from a computer or a smartphone.

The implementation of a home automation management system with the *Node-Network* extension can be an effective solution to automate processes. *Node-Network* is an open source visual programming platform that allows to create automated workflows by connecting different “nodes” or code blocks [19]. This can be useful for the particular case of automation of processes in swimming pools, since it allows the creation of automated workflows to control energy consumption, schedule pool cleaning, control the climate, among others. In addition, *Node-Network* also allows the integration of different devices and sensors through communication protocols such as *MQTT* or *HTTP*, allowing real-time data to be collected and used to optimize automated processes.

### 2.2. Description of Equipment

The following equipment is available in the swimming pool system.

(A) Hydraulic drive pumps (Figure 3a). Two pumps are used. A 1 HP pump used in the swimming pool filtration systems to move and filter the pool water. This pump is responsible for purifying the pool water on a daily basis during the swimming season, which helps to keep the water clean and clear. The second one is a 150 W pump responsible for circulating the water through the heating element. Hydraulic pumps are robust and reliable and can operate for long periods of time without maintenance.
(B) Electric heater resistance (Figure 3b). This is an 18 kW electrical resistance used as one of the alternatives to heat the swimming pool water. An electrical resistance heats up when electric current is applied to it, and transfers its thermal energy to the air or water circulating around it. Electric heaters of this type are common in residential and commercial heating systems, and can be used in both indoor and outdoor environments.
(C) Intelligent 4-channel relay (Figure 3c). This is a smart relay that connects to a Wi-Fi network and allows independent control of up to four electrical devices connected to the relay channels. This allows users to remotely control connected devices, which can be useful in a variety of applications, such as controlling lights, appliances, irrigation systems, etc. This relay is easy to set up and use, and can be controlled via a mobile app or web interface. The technical features of this device are, input voltage: ac 85~250 V, maximum current: 2200 W/10 A for each channel, WIFI standard: IEEE 802.11b/G/N (supports 2.4 GHz) + Bluetooth, working temperature accuracy: −20~70 °C, operating humidity: ≤80%.
(D) Smart temperature sensors (Figure 3d). They are used to measure the temperature of an ambient or liquid. Smart temperature sensors typically have Wi-Fi communication to transfer readings to a Wi-Fi network, allowing users to access the data remotely. In the heating option of the solar collectors, a smart temperature sensor is used to measure the temperature of the water circulating through the collectors and a second sensor is used to measure the temperature of the swimming pool water itself. The technical characteristics of this device are: power supply voltage: ac 110–230 V, temperature range from −40 °C to 99 °C, temperature measurement accuracy: ±1 °C (−40 °C, 70 °C, temperature sensor: DS18B20.
(E) Solar collectors with a total heating surface of 14 m^2^ (Figure 3e). Their mission is to collect and absorb solar energy and transfer it to the water circulating inside the collectors. The total heating surface area of a solar collector refers to the total area of the collector that is exposed to solar radiation, and is an important factor in determining the amount of energy that the collector can collect and transfer. In our case the total heating area is 14 m^2^ which indicates a moderate solar energy collection and transfer capacity.

### 2.3. Scenarios Evaluated

This section discusses the evaluation of three different heating scenarios to increase the pool water temperature by 2 degrees, from 25 °C to 27 °C (due to the drop in temperature during the night). The focus is to evaluate the energy cost associated with each scenario, with the goal of determining the most efficient way to heat the pool water. It is important to mention that energy cost refers not only to energy consumption, but also to energy efficiency and cost-benefit ratio.

#### 2.3.1. Scenario 1

In this scenario, the heating system is formed by a 18 kW electrical resistance working together with the circulating water pump.

**Figure 4:**
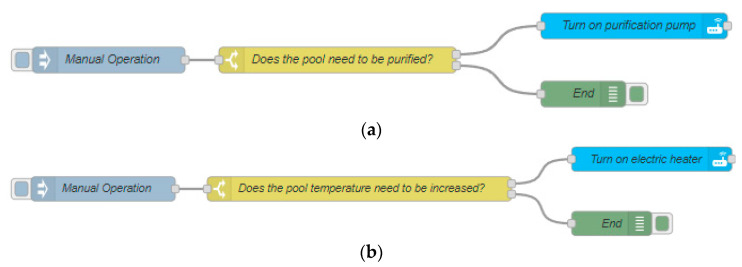
(**a**) Purification flowchart. (**b**) Swimming pool heating flow chart.

The mode of operation of this scenario is shown in the flow diagrams in Figure 4. This scenario is commonly used in residential pools and is considered as a traditional method of pool water heating. However, this scenario is characterized by a very high cost due to the high energy consumption.

Figure 4a shows the flow chart of the operation method for carrying out pool water purification. This purification process is carried out daily with a purification time of 90 min per day. To determine the energy cost and, therefore, the economic cost, the following Equation (1) is used. It is important to mention that pool water purification is essential to maintain good water quality and to prevent health problems.

(1) $$Consumption_{electrical}=Time_{Operating}*Power_{Sewage\;pump}$$

Similarly, the economic cost is calculated using Equation (2). It is important to mention that the economic cost is an important indicator to evaluate the feasibility of a project or a specific scenario. It should be considered in relation to the benefits obtained, such as energy savings and improved pool water quality.

(2) $$Cost_{Economic}=Consumption_{electrical}*Price_{Electricity}$$

On the other hand, Figure 4b shows the process used to heat the water and reach the appropriate pool temperature. The energy consumption can be obtained using Equation (3).

(3) $$Consumption_{electrical}=\left(T_{target}-T_{actual}\right)*Heat_{Specific}*Volume_{Water}$$

As in the case of purification, where Equation (2) was used to calculate the economic cost, in the case of electrical heating for the pool, the same equation could be used to evaluate this cost.

#### 2.3.2. Scenario 2

**Figure 5:**
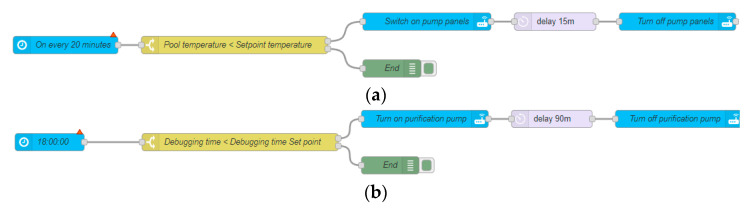
(**a**) Flow chart of solar collector pump by time. (**b**) Time-based debugging flowchart.

The second scenario replaces the electrical heating resistance with solar collectors and incorporates a temporary programming of the two operations performed daily: purification and heating. The flow charts in Figure 5a,b show the processes.

The circulating water pump is switched intermittently every 15 min during hours of solar radiation (between 11 a.m. and 18 p.m.). The duration of each start is 5 min (time necessary for the temperature of the solar collector to reach a sufficiently high value).

The daily cycle of purification of 90 min is carried out after the heating period (after 18 p.m.).

To determine the energy consumption in this scenario, Equation (4) is used.

(4) $$Consumption_{Electrical}=Time_{Operating}*Power_{Solar\;collector\;pump}$$

To determine the economic cost of this process, Equation (2) is used, this allows a direct and consistent comparison of the results obtained in the three scenarios.

#### 2.3.3. Scenario 3

**Figure 6:**
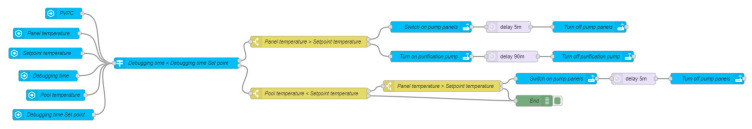
Optimization flowchart of the solar collector pump and the purification pump.

The scenario 2 is improved by incorporating temperature sensors and logic in the existing home automation system. The heating process is automated by a permanent measure of the water temperature in the solar collector and the pool water temperature (Figure 6). In this way, whenever the temperature of the solar collector is higher than that of the pool, it turns on the pump and circulates the water. Similarly, if the daily purification time has not been reached, the purification pump will be used to circulate the water instead of the solar collector pump, thus combining the two processes with the corresponding energy and economic savings.

Subsequently, in case the debugging time is not completed, the domotic manager will execute the remaining debugging time at the moment when the energy price (which is changing hour by hour and day by day) is the lowest possible. One of the integrations that offers Home Assistant is with ESIOS, which is the Spanish electricity market operator. ESIOS provides information on the real-time price of electricity which is updated every 30 min. ESIOS is integrated in Home Assistant through a particular component which is available in the Home Assistant integrations repository. The component requires an API key, which can be obtained by creating an account on ESIOS’s website.

In addition, with the logic shown in Figure 6, an intelligent management is performed so that when there is a reduction in radiation on the solar collectors and therefore the temperature decreases, the stop action of the water circulation pumps is executed.

## 3. Results

This section presents the results obtained in each of the scenarios to compare the peculiarities. The economic assessment of the cost of the equipment in each of the scenarios refers to the evaluation of the total cost of the equipment required in each scenario. This includes the cost of acquiring the devices, as well as any additional expenses associated with their installation and configuration. The economic assessment of the cost of the equipment may also include an estimate cost of the energy associated with each scenario, which allows for the calculation of the energy savings that can be achieved with the use of the equipment. With this information, it is possible to determine the payback period for each scenario, which indicates how long it will take to recover the initial cost of the equipment through the energy savings generated.

### 3.1. Scenario 1

In this scenario, the switching on of the equipment responsible for the purification of the pool water is done manually and has a defined duration of 90 min per day. The schedule used in this case is presented in Figure 4a. In addition, Figure 4b also shows the programming for heating the pool to the proper temperature. On the other hand, the process of heating the pool water is essential to ensure an adequate temperature for its use. In this scenario, the focus is on performing the purification and heating of the pool manually through specific programming. The results of the energy and economic cost of this scenario are obtained by applying Equations (1)–(3) shown above and the technical characteristics of each of the equipment installed in this scenario can be seen in Table 1. It presents the results in a clear and concise manner, which facilitates the comparison and evaluation of the results obtained in this scenario in relation to the other scenarios.

It is evident that most of the consumption is due to the pool water heating process, therefore, this is the process to be optimized energetically through the installation of other equipment and the sensorization of the installed devices in order to be able to use them. Table 2 presents the costs related to the acquisition of the equipment necessary for the purification and heating of the pool in scenario 1, in which the purification pump, the electric heater and the 4-channel relay are acquired. This table is a useful tool to evaluate the costs associated with the acquisition of the equipment necessary to carry out the pool purification and heating process in this specific scenario. In addition, this table allows to compare the costs between the different scenarios and make informed decisions about the equipment to be purchased in each case.

### 3.2. Scenario 2

**Figure 7:**
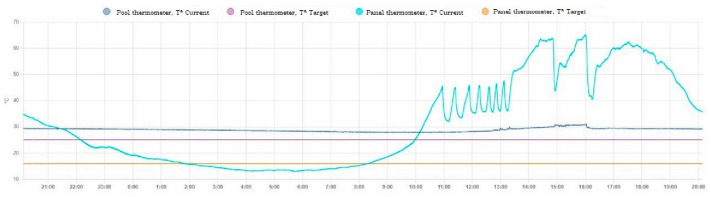
Evolution of water pool and solar collector temperature in scenario 3.

**Figure 8:**
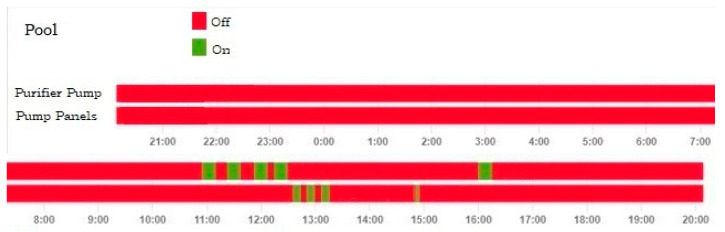
Pump panels pump and purifier pump start-up time for scenario 3.

In this scenario, 14 m^2^ of solar collectors are installed to increase the pool water temperature by solar radiation. Several smart devices must be installed to be able to act on the solar collector pumps and the purification pump. Using the programming shown in Figure 5a, the solar collector pump is activated. This logic only works in a time interval when there is solar radiation on the solar collectors, but it is not always appropriate to turn it on continuously as there may be times of cloudiness that might not heat the pool, so more time is used in scenario 2 than in the scenario that will be presented later called scenario 3. This scheduling is an important tool to maximize the use of solar radiation to heat the pool water efficiently. Figure 5b shows the schedules that allow the debugging to be turned on and off on a daily basis. The schedules in Figure 5a,b can be seen in Figure 7 and Figure 8 between 11:00 a.m. and 12:30 p.m., where this schedule was running and achieving the corresponding pool operations. As in the previous scenario, the energy and economic comparison of this scenario is presented in Table 3 and it can be observed that the processes to be carried out daily are being optimized.

In this scenario the consumption of heating the pool on a daily basis (1.84 kWh) is only 50% more than the consumption of purifying the pool (1.10 kWh), a substantial improvement has been achieved by having intelligent devices and sensors in the installation.

Table 4 shows the costs related to the acquisition of the necessary equipment for the purification and heating of the pool for scenario 2 in which the purification pump equipment, the solar collector, and the 4-channel relay are acquired.

### 3.3. Scenario 3

This scenario incorporates the installation of temperature sensors in the swimming pool and the solar collectors. A programming logic has been implemented in the intelligent devices based on the measurements provided by the sensors. Figure 6 shows the routine flowchart that allows an adequate management of the heating of the swimming pool based on the temperature comparison of the solar collector and the swimming pool water. The heating process is carried out only in the moments in which the condition is favorably fulfilled. This means that it is achieved not only in the periods when there may be solar radiation, but when an adequate solar collector temperature is reached.

Figure 7 and Figure 8 show how the programming described above has come into operation in different time periods between 12:30 and 13:30.

On the other hand, there may be times when there is already a suitable pool temperature and therefore a blocking command must be sent to prevent the start of the pump. This feature can be seen in Figure 7 and Figure 8. At 18:00 as the target temperature is reached, even though the solar collector temperature was high, the heating pump was blocked and did not work.

Finally, to optimize the daily water purification process, if the cycle has not totally been executed, the remaining filtration time is carried out in the time slots with the lowest energy cost. This is done by a communication with the database of the electricity market operator. Figure 7 and Figure 8 show how this situation occurs at 16:00 and executes the remaining filtration time to finish the daily cycle.

As shown in the previous scenarios, Table 5 shows the energy and economic comparison of scenario 3 on a daily basis.

Table 6 shows the costs related to the purchase of the equipment necessary for the purification and heating of the pool for scenario 2 in which the purification pump equipment, the solar collector, the thermostats for temperature control, and the 4-channel relay are purchased.

## 4. Discussion and Conclusions

From the different scenarios shown in Section 3, the scenario with the highest energy consumption can be determined and the saving percentages for each scenario and process can be detailed. It can be seen how both the energy consumption factor and the economic factor are optimized as numerous sensors are installed and the scheduling logic is improved in each scenario.

Table 7 shows the grouping of the consumption of each of the scenarios for the summer season (90 days), which is the time of use of the pools. It can be determined that the scenario with the highest energy consumption and, therefore, the highest economic cost is scenario 1.

The addition of the installation of the solar collectors and smart drive devices reduces the energy and economic consumption by more than 90%.

The installation of the different sensors in scenario 3 allows to improve the programming logic and to optimize the process reducing the energy consumption with respect to scenario 2 by 25% and in economic terms by more than 40% as shown in Table 7; moreover, Mohsenian-Rad et al. in their research obtained an adequate management of the different processes as detailed in this article [25].

From the data that can be found in Table 2, Table 4, Table 6 and Table 7, the corresponding return periods can be obtained for each of the scenarios proposed for analysis. The return period for scenarios 2 and 3 is almost 2 months of use of this technology since the computed time of use of this installation is 3 months. There is no clear difference between the return periods of scenarios 2 and 3, but better scheduling is available in scenario 3 and thus avoids unnecessary switching conflicts and improves user comfort, as Xu et al. [30].

It can be concluded that the application of energy efficiency measures can significantly reduce energy consumption and economic costs in a swimming pool installation. Specifically, the installation of solar collectors and smart actuation devices can lead to a reduction in energy consumption of more than 90%, and the installation of sensors can lead to a reduction in energy consumption of 25% and an economic reduction of more than 40% compared to the scenario without these measures. In addition, it appears that the payback period for these energy efficiency measures is relatively short, with a payback period of almost 2 months based on a 3-month use of the installation. Finally, it appears that the implementation of these measures may also improve scheduling and user comfort.

From the information provided in the text, the following general conclusions can be drawn:

- The implementation of energy efficiency measures can significantly reduce energy consumption and economic costs in a facility with swimming pools.
- The installation of solar collectors and smart actuation devices can lead to a very high reduction in energy consumption.
- The installation of sensors can improve the scheduling logic and optimize the process, resulting in a substantial reduction in energy consumption and a higher reduction in economic terms compared to scenarios without such features.
- Implementation of these measures can also improve scheduling and user comfort.

There are several potential lines of research that could be developed from the information provided in this research. It would be interesting to conduct a more detailed analysis of the costs and benefits of implementing energy efficiency measures such as solar collectors and smart actuation devices in swimming pool facilities. This could include an analysis of the initial installation costs as well as the long-term costs and benefits of reduced energy consumption. Moreover, it would be useful to compare the effectiveness of different energy efficiency measures in reducing energy consumption in pool facilities. This could include comparing the use of solar collectors with other renewable energy sources, or comparing the effectiveness of different types of smart actuation devices.

## Figures and Tables

**Table 1:** Diary energy consumption and economic cost of each process under scenario 1.

Process	Energy Consumption (kWh)	Economic Cost (€)
Purification	1.10	0.17
Pool heating	109.00	16.36
Total	110.10	16.5

**Table 2:** Cost of installed equipment scenario 1.

Equipment	Cost (€)
Purification pump	169.00
Pump impulsion	50.00
Electric heater	971.00
Relay 4 channels	21.00
Total	1211.00

**Table 3:** Daily energy consumption and economic cost of each process in scenario 2.

Process	Energy Consumption (kWh)	Economic Cost (€)
Purification	1.10	0.17
Pool heating	1.84	0.28
Total	2.95	0.44

**Table 4:** Cost of installed equipment scenario 2.

Equipment	Cost (€)
Purification pump	169.00
Pump impulsion	50.00
Solar collector	630.00
Relay 4 channels	21.00
Total	870.00

**Table 5:** Daily energy consumption and economic cost of each process in scenario 3.

Process	Energy Consumption (kWh)	Economic Cost (€)
Purification	1.10	0.09
Pool heating	1.10	0.17
Total	2.20	0.25

**Table 6:** Cost of installed equipment scenario 3.

Equipment	Cost (€)
Purification pump	169.00
Solar collector	630.00
Relay 4 channels	21.00
Wi-Fi thermostats	34.00
Total	854.00

**Table 7:** Comparison of energy consumption and economic cost of the different scenarios.

Process	Energy Consumption (kWh)	Economic Cost (€)
Scenario 1	9912.96	1486.95
Scenario 2	264.96 (−97.33%)	39.75 (−97.33%)
Scenario 3	198.72 (−25.00%)	22.85 (−42.50%)

## Data Availability

No new data were created or analyzed in this study. Data sharing is not applicable to this article.

## References

[1] Kraus S., Roig-Tierno N., Bouncken R.B. (2019). Digital innovation and venturing: An introduction into the digitalization of entrepreneurship. Rev. Manag. Sci..

[2] Sivaraman V., Gharakheili H.H., Fernandes C., Clark N., Karliychuk T. (2018). Smart IoT Devices in the Home: Security and Privacy Implications. IEEE Technol. Soc. Mag..

[3] Actualización de las Perspectivas de la Economía Mundial. IMF.

[4] Consumo de Energía en España Consumo Eléctrico. GuiaEnergia.

[5] Jefatura del Estado *Corrección de Errores de la Ley 7/2021, de 20 de Mayo, de Cambio Climático y Transición Energética*; [En línea]; Madrid, Spain, 2021; Volume BOE-A-2021-11870, p. 84900. https://www.boe.es/eli/es/l/2021/05/20/7/corrigendum/20210716.

[6] Jefatura del Estado *Ley 7/2021, de 20 de Mayo, de Cambio Climático y Transición Energética*; [En línea]; Madrid, Spain, 2021; Volume BOE-A-2021-8447, pp. 62009-62052. https://www.boe.es/eli/es/l/2021/05/20/7.

[7] IEA—International Energy Agency. https://www.iea.org.

[8] IDAE Consumos del Sector Residencial en España. [En línea]. https://www.idae.es/informacion-y-publicaciones/estudios-informes-y-estadisticas.

[9] European Commission, Directorate General for Communication (2015). Energía: Energía Sostenible, Segura y Asequible para los Europeos.

[10] Stojkoska B.L.R., Trivodaliev K.V. (2017). A review of Internet of Things for smart home: Challenges and solutions. J. Clean. Prod..

[11] Tsai K.-L., Leu F.-Y., You I. (2016). Residence Energy Control System Based on Wireless Smart Socket and IoT. IEEE Access.

[12] Hugo Marcelo T.S., Gabriel Eduardo M.P., Christopher Junior P.A. Implementation of a low cost smart home based on standard 802.11 b/g/n WiFi. Proceedings of the 2019 7th International Engineering, Sciences and Technology Conference, IESTEC 2019.

[13] Seydoux N., Drira K., Hernandez N., Monteil T. (2016). IoT-O, a Core-Domain IoT Ontology to Represent Connected Devices Networks. Knowledge Engineering and Knowledge Management.

[14] Lin H., Bergmann N.W. (2016). IoT Privacy and Security Challenges for Smart Home Environments. Information.

[15] Xu B., Hussain B., Wang Y., Cheng H.C., Yue C.P. (2022). Smart Home Control System Using VLC and Bluetooth Enabled AC Light Bulb for 3D Indoor Localization with Centimeter-Level Precision. Sensors.

[16] Longo C.F., Santoro C., Santoro F.F. Meaning Extraction in a Domotic Assistant Agent Interacting by Means of Natural Language. Proceedings of the 2019 IEEE 28th International Conference on Enabling Technologies: Infrastructure for Collaborative Enterprises, WETICE 2019.

[17] Sayed A., Himeur Y., Alsalemi A., Bensaali F., Amira A. (2021). Intelligent Edge-Based Recommender System for Internet of Energy Applications. IEEE Syst. J..

[18] Abrishamchi M.A.N., Zainal A., Ghaleb F.A., Qasem S.N., Albarrak A.M. (2022). Smart Home Privacy Protection Methods against a Passive Wireless Snooping Side-Channel Attack. Sensors.

[19] Abdelouhahid R.A., Debauche O., Mahmoudi S., Marzak A., Manneback P., Lebeau F. Open Phytotron: A New IoT Device for Home Gardening. Proceedings of the 2020 5th International Conference on Cloud Computing and Artificial Intelligence: Technologies and Applications (CloudTech).

[20] Park W.-K., Choi C.-S., Lee I.-W., Jang J. (2010). Energy efficient multi-function home gateway in always-on home environment. IEEE Trans. Consum. Electron..

[21] Suh C., Ko Y.-B. (2008). Design and implementation of intelligent home control systems based on active sensor networks. IEEE Trans. Consum. Electron..

[22] Patel K.K., Patel S.M., Scholar P. (2016). Internet of things-IOT: Definition, characteristics, architecture, enabling technologies, application & future challenges. Int. J. Eng. Sci. Comput..

[23] Velasco-Álvarez F., Fernández-Rodríguez Á., Ron-Angevin R. (2022). Brain-computer interface (BCI)-generated speech to control domotic devices. Neurocomputing.

[24] Tompros S., Mouratidis N., Draaijer M., Foglar A., Hrasnica H. (2009). Enabling applicability of energy saving applications on the appliances of the home environment. IEEE Netw..

[25] Mohsenian-Rad A.-H., Wong V.W.S., Jatskevich J., Schober R., Leon-Garcia A. (2010). Autonomous Demand-Side Management Based on Game-Theoretic Energy Consumption Scheduling for the Future Smart Grid. IEEE Trans. Smart Grid.

[26] Almughram O., Ben Slama S., Zafar B. (2022). Model for Managing the Integration of a Vehicle-to-Home Unit into an Intelligent Home Energy Management System. Sensors.

[27] Barbato A., Capone A., Carello G., Delfanti M., Merlo M., Zaminga A. House energy demand optimization in single and multi-user scenarios. Proceedings of the 2011 IEEE International Conference on Smart Grid Communications, SmartGridComm 2011.

[28] Cabrera J., Mena M., Parra A., Pinos E. Intelligent assistant to control home power network. Proceedings of the 2016 IEEE International Autumn Meeting on Power, Electronics and Computing, ROPEC 2016.

[29] Esquicha-Tejada J., Copa-Pineda J. (2020). Integración de un Sistema IoT—Sistema Fotovoltaico que Permita Optimizar el Consumo del Agua Potable en el Regado de Jardines de la Ciudad de Arequipa. http://laccei.org/LACCEI2020-VirtualEdition/meta/FP212.html.

[30] Cho M.E., Kim M.J. (2022). Smart Homes Supporting the Wellness of One or Two-Person Households. Sensors.

